# Academy of Stomatology

**Published:** 1901-06

**Authors:** 

**Affiliations:** Academy of Stomatology


### ACADEMY OF STOMATOLOGY.

A regular monthly meeting of the Academy of Stomatology of Philadelphia was held at the rooms of the Academy, 1731 Chestnut Street, Philadelphia, on the evening of March 26, 1901, the President, Dr. J. T. Lippencott, in the chair.

### INCIDENTS OF PRACTICE.

*The President.*—The evening is, by recommendation of the Council, to be devoted to incidents of practice, and such are now in order.

*Dr. Gardiner.*—I would like to mention a process of immediate root filling suggested to me by Dr. Nichol, which for the last few months has given me increasing satisfaction. It is applicable both to the filling of canals from which the intentionally devitalized pulp has been removed and to cases of putrescent pulps and abscess either with or without fistula. The procedure is practically the same in all cases. After first removing the pulp and the *débris* thoroughly, the canal is reamed to, but not through, the apex with a Gates-Glidden or other similar drill, thus removing that portion of dentine next to the pulp which contains the more animal matter. Formalin is now applied to sterilize the root, which is then dried out and filled. This procedure requires but a little time in a single-rooted tooth, probably from a half to three-quarters of an hour. In one with three canals it would, of course, take a correspondingly longer time, usually about an hour. I have tried the method in some of the worst cases of putrescence which I have had, in order to thoroughly test the method, and I was particularly gratified with the treatment of a case of blind abscess which came in recently. I tapped the tooth, and there was free discharge of pus and serum. Two days later, there being no discharge, I reamed out the canals to the apex, thoroughly sterilized them and filled the canals. The tooth has remained thoroughly comfortable. I had the same result with a case where the pulp was dying. It had given considerable trouble and had been filled temporarily, but it had now reached the point where there was no doubt about the ultimate death of the pulp. I opened the tooth and removed the pulp with vapocaine, and filled the canals to the apex, after reaming them out and sterilizing. The patient was perfectly relieved of all discomfort.

(Replying to Dr. Huey.) I have been using the forty per cent. formaldehyde in full strength, but take care not to saturate the canals, simply moisten and wipe them out so as not to have any excess. So far I have had no trouble. I think one might have trouble very readily by the too free use of it. I think that five per cent. of formalin would be strong enough for all purposes, but I have been using forty per cent. simply as a matter of experiment. I have, however, used it with extreme care, and would not recommend it. I thoroughly dry the canals before filling with gutta-percha to the apex. I have been testing this method now for several months, and so far I have not had a single case that was not satisfactory in every way. I would say that this method of immediate canal filling has given me more satisfaction than any new process which I have adopted or tested for a great many years.

*Dr. Roberts.*—I wish to speak of the immediate removal of pulps by means of pressure anæsthesia, and, as the thing is new, will give to you my first and second experiences. In the first I had I went ahead very slowly, and it took me an hour to obtund, get the pulp out, treat the root thoroughly, and fill it with a temporary cotton dressing. In the second case, which was in the same mouth, after getting the rubber dam on it took me eight minutes to open into the tooth and remove the pulp without any pain to the patient beyond that from the first action of the ethyl chloride, used to benumb the pulp in order to get an exposure. In another case, in which fortunately there was no hemorrhage, I had removed the pulp and filled the root in eighteen minutes.

I have had but one failure, I believe, and I could not understand that. I could bring a great deal of pressure on the unvulcanized rubber, but could not enter the tooth, though I had a free exposure. I made an application of arsenic afterwards, but I could not find the pulp or any sign of it. Consequently I do not know what caused pain, or what it was that was sensitive.

Five per cent. formalin is as strong an antiseptic as is necessary, as any one who has had a toothache coming from formalin placed upon an exposed pulp will agree. When formalin is put, for instance, into the posterior nares or upon a wounded surface, we are reminded of our boyhood days when we robbed the wasp-nests.

I have filled immediately after removing the pulp and also after introducing a dressing of carbolic acid on cotton, and unless there is some special reason for immediate filling, I would prefer,

29

where the pulp has been freshly removed, to place a dressing of carbolic acid, so that the wound at the apex may heal. It may be unnecessary.

*Dr. Gaskill.*—In experimenting with the pressure method I find cocaine to be unnecessary. The anæsthesia is from the pressure and not from the cocaine. It can be done with absolute alcohol only. Simply make pressure, and the same results are obtained. In the first case tested, I had the pulp out in two minutes. Of course, that was a very accessible tooth,—a lateral with a large cavity. In the other cases, where there was no cocaine used, the results were just as satisfactory; in other words, four or five minutes were required to remove the pulp.

*Dr. Kirk.*—It is a notorious fact that in dentistry, as well as in every other profession, ideas seem to diffuse slowly, very slowly in some cases. It is rather interesting to me to-night to hear this discussion of formalin, when I remember that it was eight or nine years ago that Dr. Mascort, of Paris, came into my office and offered me an ounce vial filled with "formol," which is, like "formalin," a forty per cent. solution of formaldehyde in water, and told me that in one of the hospitals in Paris this preparation, diluted, had been used indiscriminately and with uniform success by one of the medical or surgical staff for the treatment of cases of toothache, no matter whether they came from exposed or putrescent pulps. Within a few days after getting my preparation from Dr. Mascort I divided the bottle with Dr. Jack, and told him the story, and he tells me just now that he has been using formalin in his practice for this purpose more or less ever since. It seems to me that it is an ideal substance for the purpose which has been described here to-night. It has certain properties which make it extremely valuable. In the first place, it is an excellent germicide. In the second place, it has a peculiar tanning effect on albuminous or gelatinous matter. Thirdly, it is volatile at the ordinary body temperature, and will expand beyond the apex and bring about sterilization of the canal and, to a limited degree, of the periapical region, if the preparation is properly and carefully used. When Dr. Gardiner says that he uses a forty per cent. solution with safety, I take it for granted that he uses it with extreme caution and in extremely small amounts. It is a well-known fact that formalin is horribly irritant and destructive of vital tissue, and its use is dangerous unless that fact is kept in view and unless it is used in extremely minute amounts. In the clinic at the Dental Department of the University of Pennsylvania it is used quite extensively, but in not over two per cent. solution; even a one per cent. solution is strong enough. In histological or pathological work it is one of the best fixing agents, and a five or ten per cent. solution will harden tissues very rapidly. Tissues one inch thick can be hardened in twenty-four hours with a ten per cent. solution. It may be a beneficial thing if used within the limits of safety, or a very irritating thing if used in excess.

*Dr. Gaskill.*—I have used formalin, and in several cases I found a slight periostitis the day after filling the root, but it subsided, and I have yet to recognize a failure in any case where I have used it.

(Replying to Dr. Kirk.) I use a five per cent. solution. I have simply wiped out the canal in several instances. There has been a slight soreness, but in twenty-four hours that has entirely disappeared.

*Dr. Roberts.*—I have been using a five per cent. solution for four years, and have never had any soreness following the use of it, but I have used enough on cotton to wipe out the cavity, leaving it in only a few seconds. I then wipe out and dry the canal, and fill with my canal seal. I prefer not to seal formalin in the root.

(Replying to Dr. Hickman.) After using the formalin, as a rule you do not need much antiseptic dressing. I simply use carbolic acid. It coagulates; the formalin also coagulates. I have used a five per cent. formalin root-canal dressing and kept it in for two or three days where I have failed to stop a tooth, and kept it comfortable. Where a root-canal refuses to be comfortable with any of the essential oils, it will remain comfortable under a five per cent. solution of formalin for a couple of days, and I have never had periodontitis follow its use. I have had it follow the sulphuric acid treatment.

*Dr. Huey.*—May I be allowed to digress for a moment? The question of coagulation under formalin has been brought up. I am in the habit of making polishing wheels of corundum and gelatin which is coagulated with formalin, and they are hard enough to do very good polishing in the mouth. I merely bring this up to show the power of formalin to coagulate albumin.

*Dr. Darby.*—It occurs to me that possibly it would be interesting to the members to have me repeat what I said at Old Point Comfort about a rapid method of devitalizing the pulp. Possibly you have read of what I said on that occasion, but I have brought with me the little instrument with which it is done, and will describe in the fewest words possible my method of devitalizing a pulp almost instantaneously. I first discovered this about a year ago, when I inadvertently broke off a lateral incisor, decayed on both mesial and distal surfaces. The tooth was a vital one, and the lady was going away the next day. It was necessary that I should do something. I said nothing to the lady, but took a very sharp new bur, revolved my engine very rapidly and cut through the dentine until I approached the pulp, and made a good exposure. I then touched a crystal of carbolic acid to the pulp, and it at once made a white eschar. It then occurred to me that I could inject into that pulp some chloroform and carbolic acid. I filled a syringe with equal parts of chloroform and carbolic acid. The nozzle I put into the pulp-chamber and packed a little gutta-percha stopping around it. I then attached the syringe and forced the piston down, but my patient did not wince. Instantly I twisted the pulp out with a sharp broach. It was as white as any piece of tripe or skin tissue. I said to my patient, "Did I hurt you?" She said, "Not at all." I put on a Logan crown temporarily, and sent the lady away.

In another case, for bridging purposes, I drilled into each of two sound incisors with a sharp bur, exposing them; then took a crystal of carbolic acid and carried it into my drill opening. I packed the canula in with gutta-percha. In less than three minutes I had both pulps out, and they were blanched as white as a ghost. I have done it in a half-dozen cases, and each time the patient has said, "It does not hurt in the slightest degree." I give it to you, gentlemen, for what it is worth. It is carbolic acid and chloroform, equal parts, injected by gentle pressure. This is a French syringe, a glass barrel with a glass piston. These are absolutely air-tight. There is no needle attached, simply a canula.

(Replying to Dr. Huey.) These cases were all single-rooted teeth, but I tried it in molars, and it had the same effect. You will find that your anæsthetic goes up the root with perfect ease. The method seems especially applicable to those exposures which we meet with in single-rooted teeth where you can introduce the canula a sixteenth or an eighth of an inch and pack your gutta-percha around it.

*Dr. Guilford.*—It is the general practice now to employ pressure anæsthesia, and if Dr. Darby would try that he would not wish to use a syringe again. Instead of using a special instrument and packing the gutta-percha or temporary stopping around it, he could accomplish the same result with cocaine and carbolic acid, a little unvulcanized rubber, and a ball-burnisher.

*Dr. Darby.*—Do you hurt your patient?

*Dr. Guilford.*—Only for an instant. At the first moment of pressure there is a slight sensation of pain, but after that there is none. The pulp is removed painlessly and always comes out white. There is sometimes a little hemorrhage after a moment or two, but I do not object to that; indeed, I prefer to have a little bleeding, as it tends to relieve any possible congestion. The method is perfectly simple and thoroughly effective.

*Dr. Darby.*—I would like to have Dr. Guilford tell me how, in such a case as the first one I mentioned, when there is nothing but a peg sticking down from the gum, he will be able to make pressure?

*Dr. Guilford.*—You simply put the unvulcanized rubber over the opening where you place the drug.

*Dr. Darby.*—You have no opening.

*Dr. Guilford.*—The moment you expose the pulp you make one. Dr. Gaskill tells us he has used alcohol in the same way.

*Dr. Darby.*—Where you have the cavity of decay, that is all right.

*Dr. Guilford.*—You do not need a cavity of decay, only an opening into the pulp.

*Dr. Darby.*—How much cocaine do you use?

*Dr. Guilford.*—A mass about the size of a pin-head: just as much as you can lay on the surface exposure. The rubber will produce pressure sufficient to force the medicament thoroughly into the pulp.

*Dr. Duffield.*—I have for a year been removing pulps by the method described by Dr. Guilford, but I have been using a solution of hydrochlorate of cocaine in acetic ether, which I think an advantage.

Dr. Darby stated that the society had the endorsement of the Council to listen to the remarks of Mr. J. W. Place, who possessed a new method of making seamless crowns, and that the appliances he had with him were neither patented nor patentable. It was decided to first adjourn the meeting, and then to allow Mr. Place to speak and make his demonstration.

Adjourned.

OTTO E. INGLIS,
*Editor Academy of Stomatology.*

